# Value of skull base invasion subclassification in nasopharyngeal carcinoma: implication for prognostic stratification and use of induction chemotherapy

**DOI:** 10.1007/s00330-022-08864-7

**Published:** 2022-05-31

**Authors:** Shuqi Li, Chao Luo, Wenjie Huang, Siyu Zhu, Guangying Ruan, Lizhi Liu, Haojiang Li

**Affiliations:** Department of Radiology, Sun Yat-sen University Cancer Center, State Key Laboratory of Oncology in South China, Collaborative Innovation Center for Cancer Medicine, Guangdong Key Laboratory of Nasopharyngeal Carcinoma Diagnosis and Therapy, 651 Dongfeng Road East, Guangzhou, Guangdong 510060 People’s Republic of China

**Keywords:** Skull base invasion, Nasopharyngeal carcinoma, Induction chemotherapy, Prognosis, Neoplasm staging

## Abstract

**Objectives:**

Prognoses for nasopharyngeal carcinoma (NPC) between categories T2 and T3 in the Eighth American Joint Committee on Cancer (AJCC) staging system were overlapped. We explored the value of skull base invasion (SBI) subclassification in prognostic stratification and use of induction chemotherapy (IC) to optimize T2/T3 categorization for NPC patients.

**Methods:**

We retrospectively reviewed 1752 NPC patients from two hospitals. Eight skull base bone structures were evaluated. Survival differences were compared between slight SBI (T3 patients with pterygoid process and/or base of the sphenoid bone invasion only) and severe SBI (T3 patients with other SBIs) with or without IC using random matched-pair analysis. We calculated the prognosis and Harrel concordance index (C-index) for the revised T category and compared IC outcomes for the revised tumor stages.

**Results:**

Compared to severe SBI, slight SBI showed better 5-year overall survival (OS) (81.5% vs. 92.3%, *p* = 0.001) and progression-free survival (PFS) (71.5% vs. 83.0%, *p* = 0.002). Additional IC therapy did not significantly improve OS and PFS in slight SBI. The proposed T category separated OS, PFS, and locoregional recurrence-free survival in T2 and T3 categories with statistical significance. An improved C-index for OS prediction was observed in the proposed T category with combined confounding factors, compared to the AJCC T staging system (0.725 vs. 0.713, *p* = 0.046). The survival benefits of IC were more obvious in the advanced stage.

**Conclusions:**

NPC patients with slight SBI were recommended to downstage to T2 category. The adjustment for T category enabled better prognostic stratification and guidance for IC use.

**Key Points:**

*• For nasopharyngeal carcinoma (NPC) patients in T3 category, slight skull base invasion was a significant positive predictor for OS and PFS.*

*• NPC patients with slight SBI might not gain significant survival benefits from induction chemotherapy.*

*• Downstaging slight SBI NPC patients to T2 category would make a more accurate risk stratification, improve the predicting performance in OS, and have a better guidance in the use of IC for patients in advanced stage.*

**Supplementary Information:**

The online version contains supplementary material available at 10.1007/s00330-022-08864-7.

## Introduction

Nasopharyngeal carcinoma (NPC), a type of head and neck malignancy with 129,079 newly diagnosed cases worldwide in 2018, is endemic in Southeast Asia [1]. The prognosis of patients with advanced stage NPC remains unsatisfactory [2, 3] in the intensity-modulated radiation therapy (IMRT) era. Concurrent chemoradiotherapy (CCRT) and induction chemotherapy (IC) have been introduced to reduce recurrence and metastasis [4–9]. A staging system is used to guide timely and effective treatment for patients and avoid unnecessary treatment. However, unbalanced distribution and overlapping prognosis between T2 and T3 categories were observed in the 8^th^ edition of the American Joint Committee on Cancer (AJCC) staging system for NPC [10–12]. The current National Comprehensive Cancer Network (NCCN) guidelines [13] for NPC, based on the outcomes of clinical trials depending on the current staging system, might fail to accurately guide treatment. Thus, optimization of the current T category is required.

The bone of the skull base, which is among the most commonly invaded structures during the posterosuperior extension of NPC with an incidence rate of 50–70% [14, 15], has been regarded as the landmark for the T3 category [16]. Previous studies [12, 17–22] indicated that skull base invasion (SBI) subclassification is a significant prognostic predictor for NPC. However, some of these studies [17–21] did not further explore its value in the total T staging system. Other studies [12, 22] proposed that downstaging SBI subclassification with better prognosis into T2 category contributed to a better prognostic stratification. These studies indicated that the position of SBI in current T staging system was not ideal. Adjusting the T category of SBI subclassification might settle the problem of overlapping prognosis of T2/T3 category. Two points can be further investigated in the above studies, including the consideration of the influence of confounding factors such as plasma EBV DNA level, and identification of treatment outcome related to IC for NPC patients with different SBI subclassification, which can reflect the effectiveness of previous staging systems in guiding clinical treatment. Therefore, an in-depth study concerning the survival outcomes of NPC patients undergoing IC is warranted in investigating the optimal T category of SBI subclassification.

Based on the above studies, we enrolled a large cohort of NPC patients to investigate the value of SBI subclassification in prognostic stratification and use of IC therapy, and further validate its optimal position in the current T staging system.

## Methods and materials

### Patients and follow-ups

Ethics approval was provided by the institutional ethics committees of the two hospitals. This study was conducted in accordance with the 1964 Declaration of Helsinki and the requirement of informed consent was waived due to the retrospective nature of this study.

A total of 1752 patients with pathologically confirmed NPC at Sun Yat-sen University Cancer Center (Hospital 1, *n* = 1320) and the First People’s Hospital of Foshan (Hospital 2, *n* = 432) between January 2010 and March 2014 were studied retrospectively. The enrollment criteria were diagnosis of NPC with pathological confirmation; absence of distant metastasis and other tumors at first diagnosis; pretreatment MRI examination of the head and neck regions; complete records of clinical data; and a complete course of IMRT without any dropout.

During the 5-year follow-up, patients returned to the hospitals for regular examination every 3 months during the first 2 years, and biannually thereafter. The date of the first diagnosis of NPC was set as the starting point. Overall survival (OS) was calculated as the primary endpoint; progression-free survival (PFS), distant metastasis-free survival (DMFS), and locoregional recurrence-free survival (LRFS) were calculated as the secondary endpoints.

### MRI protocol

Detailed information on the MRI protocol is presented in the Supplementary materials.

### MRI assessment and criteria for skull bone invasion

**Fig. 1:**
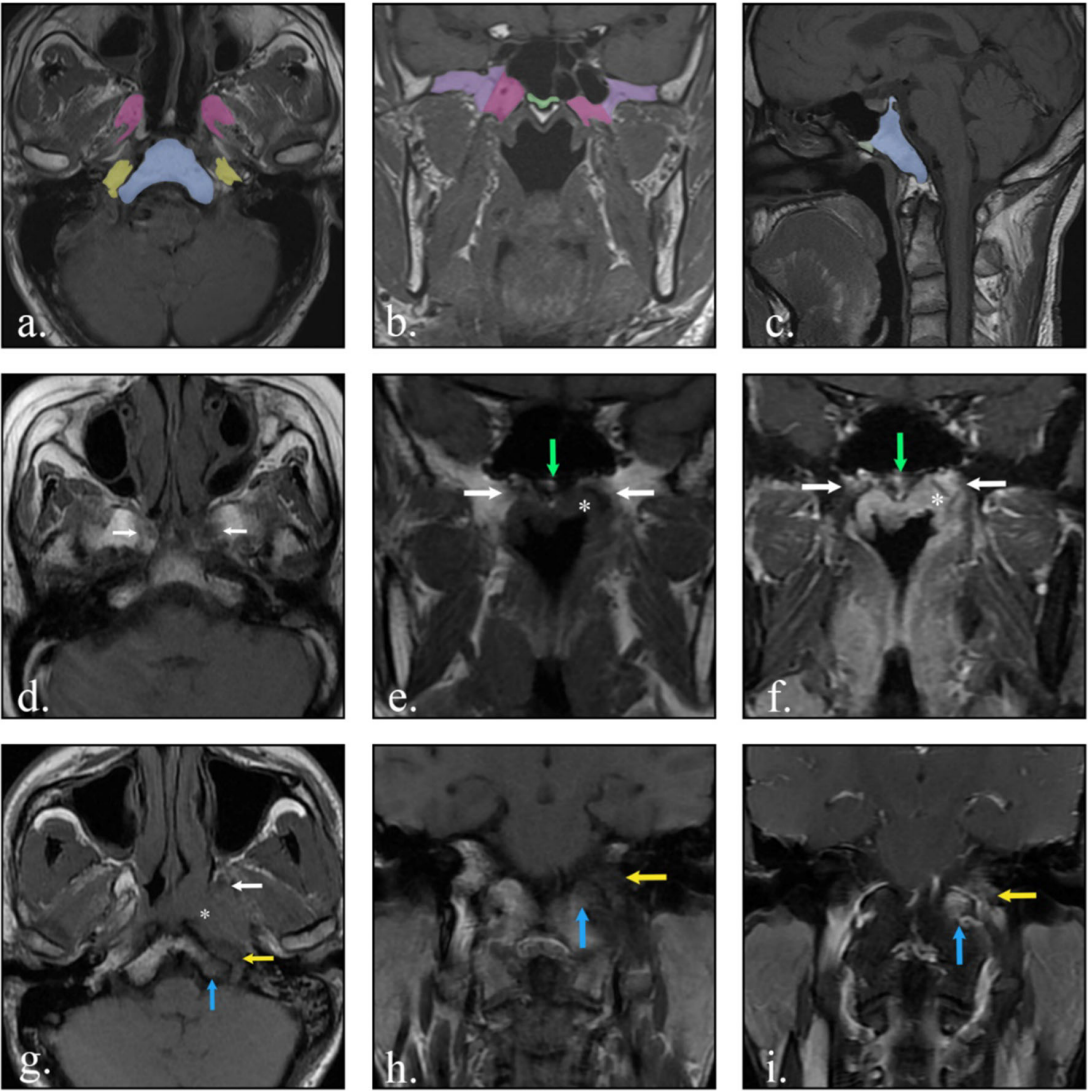
Diagram for the normal skull base bone structures, representative MRI images illustrating slight SBI and severe SBI in patients with nasopharyngeal carcinoma. Axial (**a**), coronal (**b**), and sagittal (**c**) images illustrating normal skull base bone position. Landmarks of slight SBI are pterygoid processes (pink, the bone between the extension lines of the medial and lateral pterygoid plates) and base of the sphenoid bone (green)— thesphenoid bone between the inside edge of bilateral pterygoid processes (coronal), and before the extension line of the front edge of the clivus (sagittal). Structures related to severe SBI are the petrous apexes (yellow), clivus (blue), great wings of the sphenoid bone (purple), occipital condyles, cervical spines, and paranasal sinuses. Example of NPC patients with slight SBI (**d–f**): Bilateral pterygoid processes (white arrows) and base of the sphenoid bone (green arrows) were invaded by tumor (*), with hypointense signal on axial (**d**) and coronal (**e**) T1WI, and with enhancement on fat-suppressed contrast-enhanced T1WI (**f**). Example of NPC patients with severe SBI (**g**–**i**): petrous apex (yellow arrows), clivus (blue arrows), and pterygoid process (white arrow) in the left side were invaded by tumor (*), with hypointense signal on axial (**g**) and coronal (**h**) T1WI, and with enhancement on fat-suppressed contrast-enhanced T1WI (**i**). Note 1. Occipital condyles, cervical spines, and paranasal sinuses were not painted with color

Two senior radiologists, Liu L. and Li H., with 18 and 10 years of experience in head and neck cancer respectively, evaluated MRI scans independently. Diagnostic disagreements were settled in consensus. SBI on an MRI scan was hypointense in the bone marrow on T1-weighted imaging (T1WI) and had lesion enhancement on fat-suppressed contrast-enhanced T1WI [14, 17]. The evaluated bones included the pterygoid process, base of the sphenoid bone, clivus, petrous apex, great wing of the sphenoid bone, occipital condyle, cervical spine and paranasal sinus. T3 patients were divided into slight SBI (invasion of the pterygoid process and/or base of the sphenoid bone only) and severe SBI (other SBIs) (Fig. 1). Based on our study, slight SBI represents the invasion of sphenoid bone between the lateral borders of the bilateral pterygoid processes, and before the front edge line of the clivus.

### Treatment

Treatments for 1752 patients complied with the standardized treatment protocols for NPC at hospital 1 and hospital 2, following the NCCN guidelines from 2010 to 2014. All patients received IMRT. Patients at stage II–IV were recommended to receive concurrent chemotherapy, and induction chemotherapy was optional for them according to the protocols from clinicians. The chemotherapy regimens were consistent with a previous study [12]. Among them, 302 (17.2%) received IMRT alone, 558 (31.9%) received CCRT alone, and 892 (50.9%) received IC+CCRT. In IMRT, target delineation under the International Commission on Radiation Units and Measurements Reports 50 and 62 [23, 24]. Detailed protocols for IMRT and chemotherapy are presented in the Supplementary materials. Salvage therapy, such as surgery, re-radiation, and chemotherapy, was initiated for patients who developed recurrence and persistent disease. Acute adverse events for NPC patients at hospital 1 were collected from medical records during treatments, which were graded according to the Common Terminology Criteria for Adverse Events (CTCAE) Version 4.0 [25].

### Statistical analyses

The flowchart of the study is presented in Fig. A1. Kappa values were used to evaluate consistency for SBI assessments in 184 NPC patients. The distribution differences for baseline characteristics between slight SBI and severe SBI, and distribution differences for SBI condition between the two hospitals were compared using the chi-squared test, Fisher’s exact test, and Student’s *t*-test. Plasma EBV DNA load was treated as a categorical variable [26]. Next, confounding factors with *p* values < 0.05 in the univariate analysis were incorporated into the multivariate Cox regression models to calculate hazard ratios (HRs) with 95% confidence intervals (CIs) and adjusted *p* values. Survival differences with Kaplan-Meier method were calculated between the slight SBI and severe SBI groups, and between the slight SBI group and T2 category. To evaluate the prognostic value of IC in NPC patients with different SBI subclassifications, a 1:1 random matched-pair analysis of patients with stage III/IV NPC was performed using the T category, N category, and age group to eliminate the influence of confounding factors. The classification of age group was in accordance with a previous study [12]. Acute adverse events for patients treated with and without IC at hospital 1 were compared using chi-squared test or Fisher’s exact test.

Survival differences of the T categories of the 8^th^ AJCC staging system and those of the proposed T category were compared. The Harrel concordance index (C-index) was used to evaluate the prediction performance of survival using the Hmisc package in R. In train and test cohorts, C-index was calculated with T category alone, and with the combination of confounding factors, respectively. Confounding factors related to OS including T category, N category, age, and EBV were selected from multivariate Cox regression analysis. Sex was eliminated after stepwise. Regarding treatment implications, we calculated a 1:1 matched-paired analysis for OS and PFS in NPC patients treated with or without IC for the conventional and new stage III/IV, conventional and new stage II.

The above statistical analyses were performed using R version 3.2.5 (https://www.r-project.org/) with packages, including stats, survival, rms, Hmisc, ggplot2, and survminer. Statistical significance was set at a two-tailed *p* value ≤ 0.05.

## Results

### Clinical characteristics, follow-ups, and univariate analysis

Among the 1752 patients in our study, 678 (38.70%) were initially T3 and presented with SBI without involvement of structures related to T4 category. The interobserver agreement for the diagnosis of SBI was 0.929. A total of 224 (12.78%) patients were classified into slight SBI group. The highest incidence rate of invasion was observed for the pterygoid process and base of the sphenoid bone among all SBIs in both hospitals, with total incidence rates of 50.4% and 53.9%, respectively (Table A1). The basic clinical characteristics among NPC patients at T2 and T3 category are shown in Table 1.

**Table 1:** Characteristics of patients in the T2 and T3 categories

Variables	Slight SBI (*n* = 224)	Severe SBI (*n* = 454)	*χ*^2^ *p* value^*a^	T3-total (*n* = 678)	T2 (*n* = 213)	*χ*^2^ *p* value^*b^
Age(years)			0.665			0.621
Median (IQR)	46 (38~54.2)	46 (39~54)		46 (39~54)	46 (39~55)	
Sex			0.443			0.084
Male	165 (73.7%)	347 (76.4%)		512 (75.5%)	148 (69.5%)	
Female	59 (26.3%)	107 (23.6%)		166 (24.5%)	65 (30.5%)	
EBV (1×10^3^copies/mL)			0.001^†^			0.107
< 1	124 (55.4%)	185 (40.7%)		309 (45.6%)	91 (42.7%)	
< 10	55 (24.6%)	140 (30.8%)		195 (28.8%)	77 (36.2%)	
≥ 10	45 (20.1%)	129 (28.4%)		174 (25.7%)	45 (21.1%)	
Histologic type^※^			1			0.296
WHO type 1/2	8 (3.6%)	15 (3.3%)		23 (3.4%)	11 (5.2%)	
WHO type 3	216 (96.4%)	439 (96.7%)		655 (96.6%)	202 (94.8%)	
T category‡			< 0.001^†^			< 0.001^†^
T1	0	0		0	0	
T2	0	0		0	213 (100%)	
T3	224 (100%)	454 (100%)		678 (100%)	0	
T4	0	0		0	0	
N category‡			< 0.001^†^			0.087
N0	56 (25%)	56 (12.3%)		112 (16.5%)	30 (14.1%)	
N1	121 (54%)	271 (59.7%)		392 (57.8%)	124 (58.2%)	
N2	31 (13.8%)	95 (20.9%)		126 (18.6%)	33 (15.5%)	
N3	16 (7.1%)	32 (7%)		48 (7.1%)	26 (12.2%)	
Stage‡			1			< 0.001^†^
I	0	0		0	0	
II	0	0		0	154 (72.3%)	
III	208 (92.9%)	422 (93%)		630 (92.9%)	33 (15.5%)	
IV	16 (7.1%)	32 (7%)		48 (7.1%)	26 (12.2%)	
Chemotherapy			0.058			0.157
No	35 (15.6%)	48 (10.6%)		83 (12.2%)	34 (16%)	
Yes	189 (84.4%)	406 (89.4%)		595 (87.8%)	179 (84%)	
Induction chemotherapy			< 0.001^†^			0.473
No	137 (61.2%)	194 (42.7%)		331 (48.8%)	110 (51.6%)	
Yes	87 (38.8%)	260 (57.3%)		347 (51.2%)	103 (48.4%)	
Death			0.002^†^			0.82
No	206 (92%)	378 (83.3%)		584 (86.1%)	185 (86.9%)	
Yes	18 (8%)	76 (16.7%)		94 (13.9%)	28 (13.1%)	
Distant metastasis			0.081			0.725
No	203 (90.6%)	390 (85.9%)		593 (87.5%)	184 (86.4%)	
Yes	21 (9.4%)	64 (14.1%)		85 (12.5%)	29 (13.6%)	
Locoregional recurrence			0.224			0.181
No	205 (91.5%)	401 (88.3%)		606 (89.4%)	197 (92.5%)	
Yes	19 (8.5%)	53 (11.7%)		72 (10.6%)	16 (7.5%)	
Progress			0.003^†^			0.391
No	188 (83.9%)	334 (73.6%)		522 (77%)	170 (79.8%)	
Yes	36 (16.1%)	120 (26.4%)		156 (23%)	43 (20.2%)	

*Abbreviations*: *EBV* Epstein-Barr virus, *IQR* interquartile range, *n* number of patients, *WHO* World Health Organization, *Slight SBI* T3 patients with invasion of the pterygoid process and/or base of the sphenoid bone only, *Severe SBI* T3 patients with other SBIs

*p values were calculated for characteristics distribution between ^*a^slight SBI and severe SBI, ^*b^T3 and T2 category, using Fisher’s exact test or the chi-squared test for categorical variables and Student’s *t*-test for continuous variables

^※^According to the 2005 World Health Organization classification of tumors

^†^*p* < 0.05

^‡^According to the 8^th^ edition of the AJCC staging system

In the whole cohort (*n* = 1752), after a median follow-up of 61.47 months, 387 (22.09%) patients had disease progression: 244 (13.93%) patients died, 225 (12.84%) patients had distant metastasis, and 159 (9.08%) patients experienced locoregional recurrence. The failure patterns of slight SBI and severe SBI patients are listed in Table 1.

In terms of confounding factors: age, sex, EBV DNA load, N category, and IC were related to OS; age, EBV DNA load, N category, and IC were related to PFS; EBV DNA load, N category, chemotherapy, and IC were related to DMFS; N category and IC were related to LRFS. Since treatment was determined by cancer stage, chemotherapy and IC were not regarded as the final confounding factors in the prediction model (univariate analysis: Table A2; multivariate analysis: Table 2).

**Table 2:** Multivariate analysis when exploring the prognostic value of slight SBI

	Variables	OS		PFS		DMFS		LRFS	
		HR (95% CI)^*^	*p* value^†^	HR (95% CI)^*^	*p* value^†^	HR (95% CI)^*^	*p* value^†^	HR (95% CI)^*^	*p* value^†^
T2 vs. slight SBI	Age	1.04 (1.01 – 1.07)	0.01^‡^	1.02 (1.00 – 1.04)	0.08	NA		NA	
	Female sex	1.01 (0.51 – 1.98)	0.99	NA		NA		NA	
	EBV	2.54 (1.14–5.68)	0.02^‡^	2.04 (1.15 – 3.62)	0.01^‡^	2.33 (1.03 – 5.26)	0.04^‡^	NA	
	N category^§^	8.21 (2.02 – 33.33)	0.00^‡^	3.43 (1.35 – 8.75)	0.01^‡^	4.42 (1.24 – 15.79)	0.02^‡^	2.65 (0.73 – 9.65)	0.14
	Chemotherapy	NA		NA		2.13 (0.64 – 7.03)	0.22	NA	
	IC	0.84 (0.44 – 1.61)	0.60	0.95 (0.58 – 1.56)	0.85	0.75 (0.41 – 1.38)	0.36	1.37 (0.67 – 2.82)	0.39
	Slight SBI	0.66 (0.36 – 1.21)	0.18	0.90 (0.57 – 1.41)	0.64	0.82 (0.46 – 1.45)	0.49	1.29 (0.65 – 2.54)	0.46
Severe SBI vs. slight SBI	Age	1.02 (1.01 – 1.04)	0.01^‡^	1.01 (0.99 – 1.02)	0.27	NA		NA	
	Female sex	0.56 (0.32 – 0.99)	0.05	NA		NA		NA	
	EBV	1.52 (0.92 – 2.53)	0.10	1.48 (0.97 – 2.25)	0.07	2.87 (1.56 – 5.27)	0.00^‡^	NA	
	N category^§^	5.52 (2.06 – 14.82)	0.00^‡^	3.91 (1.90 – 8.06)	0.00^‡^	5.72 (1.53 – 21.46)	0.01^‡^	6.13 (2.06 – 18.22)	0.00^‡^
	Chemotherapy	NA		NA		1.42 (0.62 – 3.27)	0.41	NA	
	IC	0.97 (0.63 – 1.49)	0.88	0.94 (0.67 – 1.32)	0.71	0.97 (0.62 – 1.54)	0.91	1.00 (0.61 – 1.63)	1.00
	Slight SBI	0.49 (0.29 – 0.82)	0.01^‡^	0.64 (0.44 – 0.94)	0.02^‡^	0.77 (0.47 – 1.27)	0.31	0.78 (0.46 – 1.32)	0.35

*Abbreviations*: *CI* confidence interval, *DMFS* distant metastasis-free survival, *EBV* Epstein-Barr virus, *HR* hazard ratio, *IC* induction chemotherapy, *NA* not applicable, *LRFS* locoregional recurrence-free survival, *OS* overall survival, *PFS* progression-free survival, *slight SBI* T3 patients with invasion of the pterygoid process and/or base of the sphenoid bone only, *severe SBI* T3 patients with other SBIs.

^*,†^HR and *p* values were calculated using multivariate Cox regression analysis

^‡^*p* < 0.05

^§^According to the 8^th^ edition of the AJCC staging system.

Note 1. Confounding factors related to prognosis were selected from variables (*p* < 0.05) in a univariate analysis shown in Table A2.

**Fig. 2:**
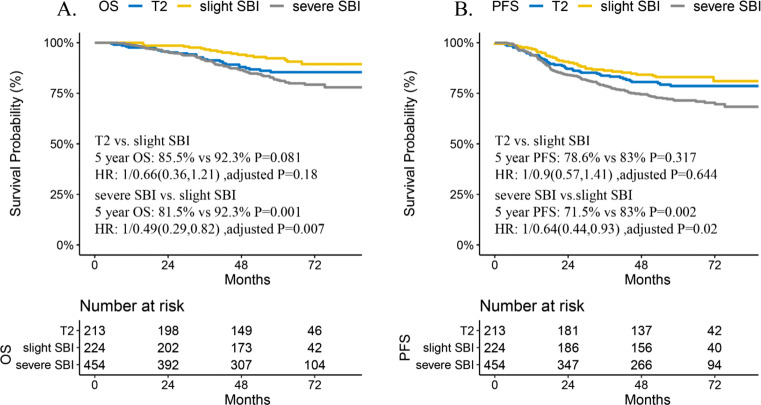
Prognosis for OS and PFS in slight SBI patients compared with T2 and severe SBI patients. Compared to severe SBI, slight SBI was a significant positive predictor of OS (**A**) and PFS (**B**) among the T3 group. Similar prognosis was observed between the slight SBI and T2 groups. Abbreviations: HR, hazard ratio; OS, overall survival; PFS, progression-free survival; SBI, skull base invasion. Note 1. Univariate analysis for testing confounding factors is shown in Table A2. Detailed result for multivariate analysis is presented in Table 2

Note 2. Survival curves are shown in Fig. 2 and Fig. A2.

### Prognostic value of SBI subclassification and treatment outcomes for IC

Compared to severe SBI, slight SBI was a statistically significant positive predictor for 5-year OS (81.5% vs. 92.3%, *p* = 0.001; HR: 0.49, 95% CI: 0.29–0.82, adjusted *p* = 0.007) and PFS (71.5% vs. 83%, *p* = 0.002; HR: 0.64, 95% CI: 0.44–0.93, adjusted *p* = 0.02). Additionally, no significant differences occurred in prognosis between the T2 and slight SBI groups (all *p* > 0.05) (Fig. 2). Improved DMFS and LRFS occurred in the slight SBI group (Fig. A2) but was not statistically significant. Detailed information on multivariate analysis is presented in Table 2.

**Fig. 3:**
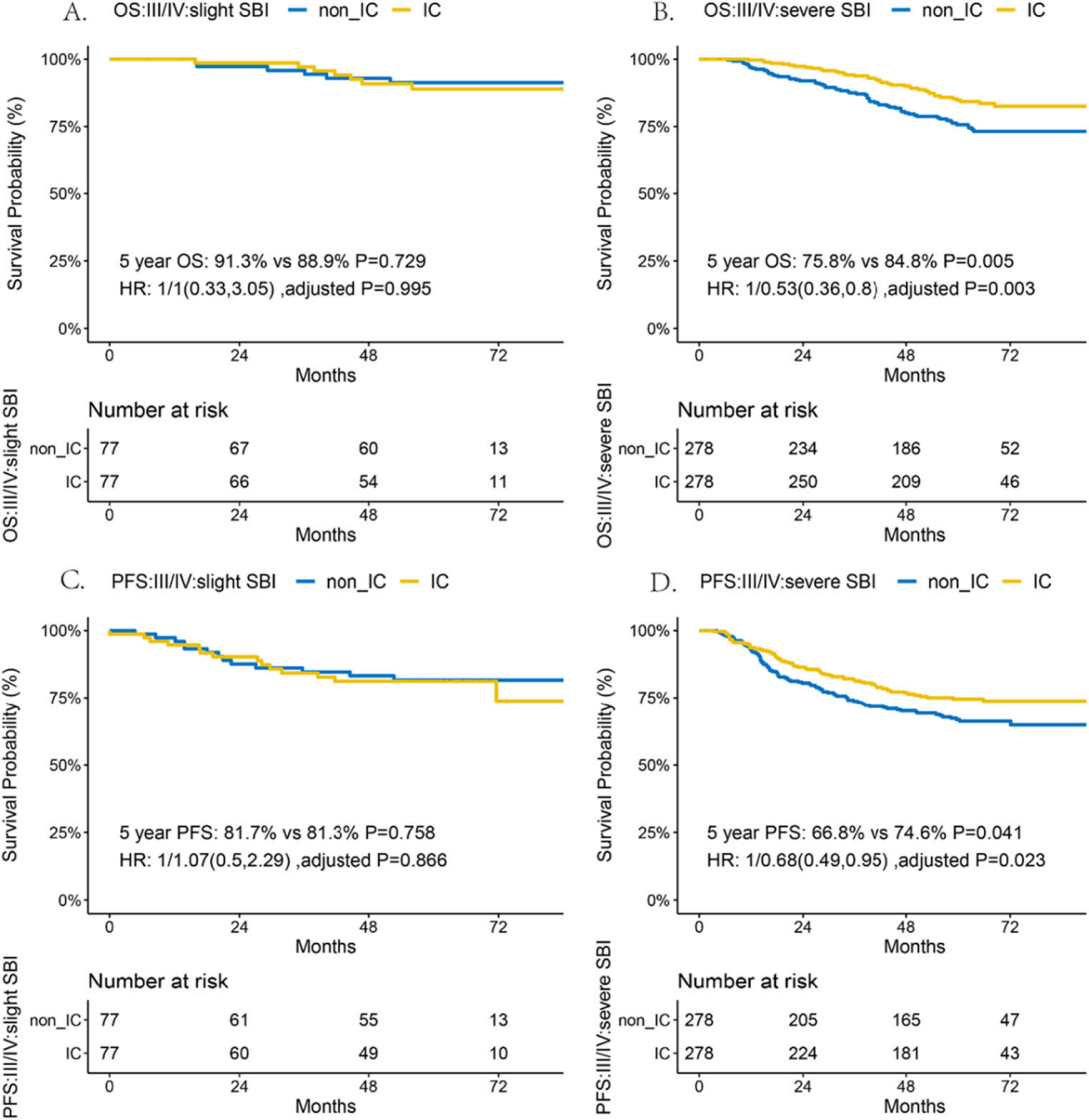
Survival outcomes for OS and PFS among SBI subclassification treated with or without induction chemotherapy. Among patients with stage III/IV NPC, slight SBI patients did not gain significant survival benefits for OS and PFS from additional IC (**A**, **C**); significantly improved OS and PFS were observed in severe SBI patients treated with additional IC than in those without IC (**B**, **D**). Abbreviations: HR, hazard ratio; IC, induction chemotherapy; OS, overall survival; PFS, progression-free survival; SBI, skull base invasion. Note 1. Basic characteristics for the pairs of slight SBI and severe SBI group are available upon request

Patients in the advanced stage were analyzed with 1:1 random matched-pair analysis. Finally, 77 pairs of patients with slight SBI and 278 pairs with severe SBI were selected. Basic characteristics of each group are available upon request. For the slight SBI group, no significant differences were observed in 5-year OS (91.3% vs. 88.9%, *p* = 0.729) and PFS (81.7% vs. 81.3%; *p* = 0.758) in patients treated with or without IC. Conversely, in the severe SBI group, patients treated with IC gained a significant survival benefit for 5-year OS (75.8% vs. 84.8%, *p* = 0.005; HR: 0.53, 95% CI: 0.36–0.8; adjusted *p* = 0.003) and PFS (66.8% vs. 74.6%,*p* = 0.041; HR: 0.68, 95% CI: 0.49–0.95, adjusted *p* = 0.023) than patients without IC (Fig. 3). Among the slight and severe SBI groups, patients receiving IC did not show significant changes in 5-year DMFS and LRFS (Fig. A3) compared to patients without IC.

For patients treated at hospital 1, the proportion of acute adverse events of patients treated with IC were significantly higher than those treated without IC in the terms of neutropenia, leucopenia, anemia, nausea, diarrhea, hair loss, digestive discomfort for total cohort, slight SBI group, and severe SBI group, respectively (*p* < 0.05) (Table A3).

### Adjustment of the T category in the slight SBI group for better prognostic prediction and use of IC

**Fig. 4:**
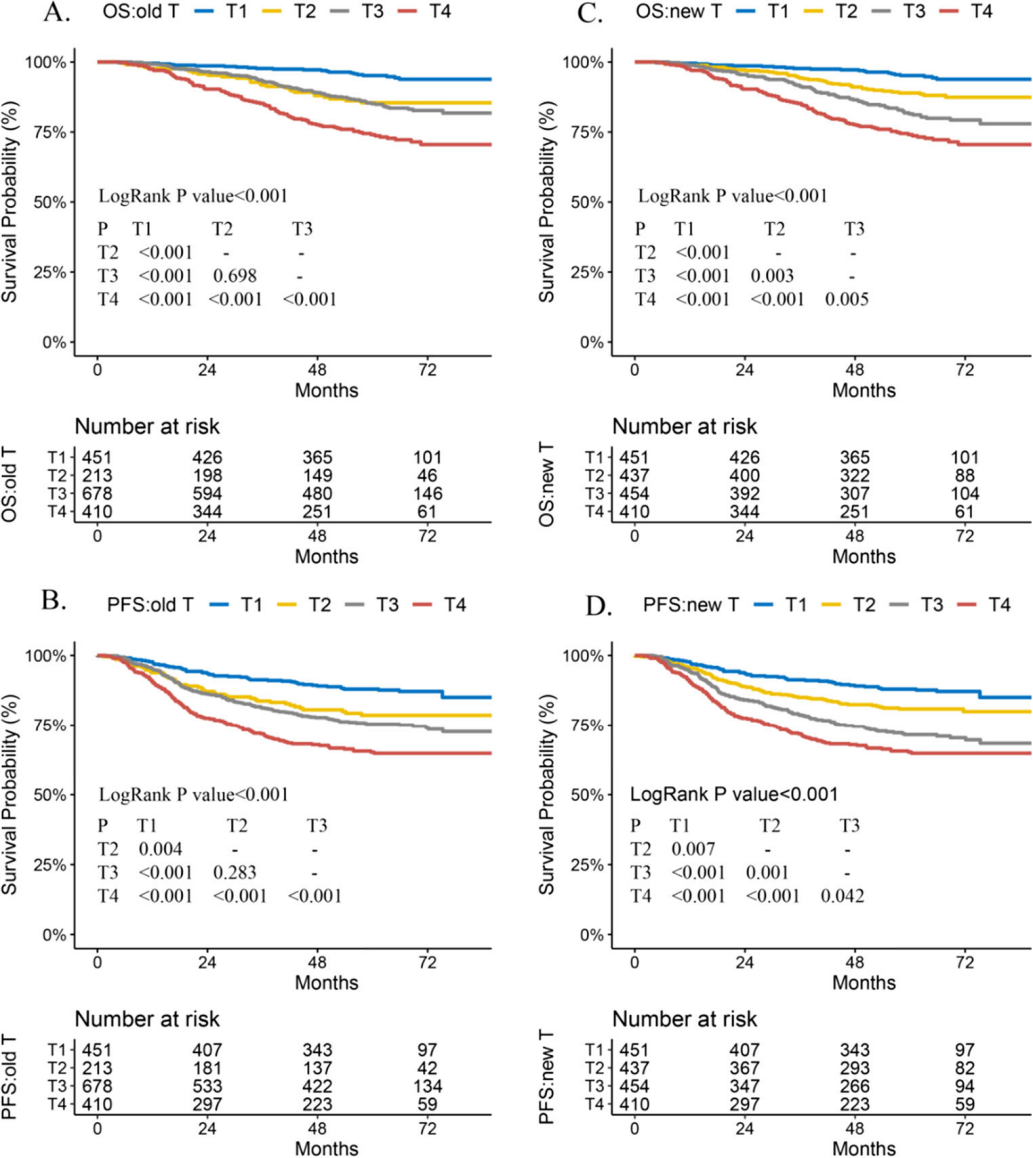
Prognosis for OS and PFS in the 8^th^ edition AJCC T staging system and proposed T category. In the 8^th^ edition AJCC T staging system, the OS (**A**) and PFS (**B**) for T2 and T3 categories almost overlapped. After downstaging slight SBI group from T3 to T2 category, significant separated prognosis was observed in the proposed T category (**C**, **D**). Abbreviations: AJCC, American Joint Committee on Cancer; HR, hazard ratio; IC, induction chemotherapy; OS, overall survival; PFS, progression-free survival; SBI, skull base invasion. Note 1. Detailed results are shown in Table A4. Note 2. The survival curves for DMFS and LRFS in the 8^th^ edition AJCC T staging system and proposed T category are shown in Fig. A4

Based on the analysis, 224 patients with slight SBI were downstaged from the T3 to the T2 category. The new distribution of the proposed T category was 437 T2 patients and 454 T3 patients. Significant survival differences in 5-year OS, PFS, and LRFS were observed between the new T2 and T3 categories (all *p* < 0.05). The proposed T category can separate the prognosis for OS and PFS by each T category with statistical significance (all adjusted *p* < 0.05) (Fig. 4, Fig. A4, Table A4).

In the train and test cohorts, models based on the proposed T category provided a significantly improved C-index in predicting OS, PFS and LRFS than models based on the 8^th^ edition T category (all *p* < 0.001). When considering confounding factors, a significantly improved C-index for OS was obtained from the model based on the proposed T staging system (0.725, 95% CI: 0.672–0.779) than that based on the 8^th^ edition T category (0.713, 95% CI: 0.658–0.768; *p* = 0.046) in the test cohort (Table A5).

Patients were redistributed in the tumor stage groups according to the proposed T category. For OS, survival benefits in patients treated with IC improved significantly and were more obvious in the new III/IV stage (76% vs. 84.1%, *p* = 0.006; HR: 0.58, 95% CI: 0.4–0.84, adjusted *p* = 0.004) than those in patients in the conventional III/IV stage (79.8% vs. 84.4%, *p* = 0.047; HR: 0.67, 95% CI: 0.47–0.94, adjusted *p* = 0.022). For PFS, survival benefits in patients treated with IC were obvious in the new III/IV stage and were close to statistical significance (68.1% vs. 74%, *p* = 0.096; HR: 0.74, 95% CI: 0.55–1.01, adjusted *p* = 0.054). NPC patients in stage II gained no survival benefit from the use of IC in both the new and conventional 8^th^ tumor stages (all *p* > 0.05) (Fig. A5).

## Discussion

In our multicenter study, we found that downstaging of the slight SBI group from T3 to T2 category yielded a balanced patient distribution, better prognosis prediction, and better guidance of IC use. The slight SBI was a significantly positive predictor for OS and PFS, consistent with previous findings [12]. Treatment outcomes from IC supported the adjustment of their T category. Additionally, the proposed T category may significantly improve the prediction ability for OS.

Currently, MRI is recommended as the main imaging examination for NPC patients during first diagnosis and follow-up [13]. It is sensitive to subtle changes in the bone marrow caused by early tumor infiltration [15, 27], resulting in early detection of SBI. During NPC extension, the incidence rate of SBI was highest among all the anatomical structures adjacent to the nasopharynx [28]. Consistent with previous findings [18–20], invasions of the pterygoid process and sphenoid base were the top two SBIs in our study, The high incidence rate may be because they were located above the nasopharynx, close to the origin site of NPC [18]. Therefore, they are easily invaded when NPC extends upward. Additionally, the lack of pharyngobasilar fascia barrier and soft tissue may facilitate infiltration, compared to the invasion of the parapharyngeal space and carotid sheath [18, 19].

Consistent with previous findings [12], survival outcomes and 5-year survival curves for OS and PFS were better in patients with slight SBI than those with severe SBI, indicating that slight SBI was a significantly positive factor for OS and PFS among T3 NPC patients. Potential reasons include tumor volume remains small in the slight SBI group when it invades the adjacent structures around the nasopharynx [12], leading to a small tumor burden with better local control [29, 30]. Severe SBI is often associated with a larger tumor size [30, 31], resulting in a higher probability of soft tissue invasion and infiltration along the skull base foramina, thus increasing the risk of distant metastasis. The slight SBI group could not gain a significant survival benefit from additional IC therapy, strongly supporting the downstaging of the slight SBI group to the T2 category. The following are the strengths of the proposed T category: The overlapping survival curves between the T2 and T3 categories of the 8^th^ AJCC staging system were resolved into significantly separated survival curves for OS, PFS, and LRFS between the new T2 and T3 categories after adjusting for confounding factors. Additionally, significant survival differences were observed in OS and PFS in each T category of the proposed T staging system, and the prediction performance for OS was improved. Moreover, the distribution of NPC patients in each T category was more balanced. Therefore, the proposed T category may yield a more accurate risk stratification.

For the current treatment of NPC patients with SBI, IC+CCRT has been recommended with 2A-level evidence [13]. The independent prognostic value of the SBI subclassification suggested that individualized treatment should be further refined. Previous studies have agreed that more intensive treatment protocols could be administered to SBI subclassification with poor prognosis but lacking further verification [20, 21]. The improved OS and PFS in the severe SBI group treated with additional IC in our matched-pair analysis proved that IC, with the potential to eradicate micrometastasis [32], is beneficial for reducing the risk of death and progression. However, in slight SBI group, no significant survival benefits were observed for those treated with additional IC. On one hand, this may be because better local control may be achieved for slight SBI group under the precise description of tumor extension from MRI [19] and individualized radiation dose distribution under IMRT [20]. The combination of concurrent chemotherapy may further improve OS [5, 7]. On the other hand, ideal regimens and treatment cycles for IC have not been established because of the inconsistent responses to IC [32–36]. For slight SBI group, IC cannot bring significant survival benefits, but accomplished with increasing incidence of acute side effects for hematological events, hair loss, and digestive discomfort, which were also reported from phase 3 randomized controlled trials [8, 9]. Avoiding additional IC in slight SBI group can reduce extra physical and economic burdens, and guide timely radiotherapy. Based on the proposed T category, we proposed a new tumor stage for the stratified use of IC. IC was recommended to NPC patients in the new III/IV stage rather than those in the new II stage, providing a reference for subsequent clinical trials. Overall, the T category adjustment for SBI subclassification contributed to patient treatment guidance.

Our study was limited in that histological confirmation of SBI could not be obtained because of the distinct anatomical position of the skull base bone. Second, to fully demonstrate the differences of acute adverse events incidence rate among treatment, more information should be collected for NPC patients at hospital 2. Third, although the N category was enrolled as a confounding factor in multivariate analysis, further research is required to evaluate the survival benefits of IC in slight SBI group with stratified analysis based on N category. Fourth, in this study, the presence or absence of vidian canal involvement (adjacent to pterygoid process) may not be a prognostic factor, which might attribute to the sub-analysis with a limited number of cases. Hence, a well-designed, prospective, randomized clinical trial for IC is warranted to validate our treatment suggestions.

Subclassification and reclassification of SBI contributed to accurate prognostic stratification and the development of individualized treatment. The slight SBI group was recommended to the T2 category for its better prognosis for OS and PFS and similar survival outcomes between patients treated with or without IC. After redistribution, the proposed T category had a high predictive ability for OS and was an indicator for the use of IC.

## Supplementary Information

ESM 1 (DOCX 12975 kb)

## Abbreviations

AJCC: American Joint Committee on Cancer
CCRT: Concurrent chemoradiotherapy
CE: Contrast-enhanced
CI: Confidence interval
DMFS: Distant metastasis-free survival
EBV: Epstein-Barr virus
FSE: Fast-spin echo
HR: Hazard ratio
IC: Induction chemotherapy
IMRT: Intensity-modulated radiation therapy
LRFS: Locoregional recurrence-free survival
NCCN: National Comprehensive Cancer Network
NPC: Nasopharyngeal carcinoma
OS: Overall survival
PFS: Progression-free survival
SBI: Skull base invasion
T1WI: T1-weighted imaging
TE: Echo time
TR: Repetition time

## Declarations

### Guarantor

The scientific guarantor of this publication is Haojiang Li, MD, and Lizhi Liu, MD.

### Statistics and biometry

No complex statistical methods were necessary for this paper.

### Methodology

• retrospective

• observational

• performed at two institutions
