# Supplementary material for: Value of skull base invasion subclassification in nasopharyngeal carcinoma: implication for prognostic stratification and use of induction chemotherapy
Source: Eur Radiol. 2022 May 31;32(11):7767–77. doi: 10.1007/s00330-022-08864-7 (PMC9668954; doi:10.1007/s00330-022-08864-7)
Supplement: Supplementary file 1 — (DOCX 12975 kb) [file 330_2022_8864_MOESM1_ESM.docx]

**Supplementary materials**

**for**

**Value of Skull Base Invasion Subclassification in Nasopharyngeal Carcinoma: Implication for Prognostic stratification and Use of Induction Chemotherapy**

1. **Supplementary methods**

MRI protocol

Treatment

1. **Supplementary tables**

Table A1. Incidence of skull base invasion among 1,752 nasopharyngeal carcinoma cases in two hospitals

Table A2. Univariate analysis of confounding factors related to prognosis

Table A3. Numbers of acute adverse events during treatment for patients with nasopharyngeal carcinoma in Hospital 1

Table A4. Prognosis in the 8^th^ edition AJCC T staging system and proposed T category

Table A5. C-index of the proposed T staging system and 8^th^ edition AJCC T staging system in patients with NPC

1. **Supplementary Figures**

Fig. A1. Flowchart of the study

Fig. A2. Prognosis for DMFS and LRFS in slight SBI patients compared with T2 and severe SBI patients

Fig. A3. Survival outcomes for DMFS and LRFS among SBI subclassification treated with or without IC

Fig. A4. Prognosis for DMFS and LRFS in the 8th edition AJCC T staging system and proposed T category

Fig. A5. The implication of the proposed T category in the use of IC

**Supplementary methods**

**MRI protocol**

Pretreatment MRI examination was performed using a 1.5-T system (Signa CV/i, General Electric Healthcare) or a 3.0-T system (Magnetom Tim Trio, Siemens). The scanning range, with a combined head and neck coil, ranged from the suprasellar cistern to the inferior margin at the sternal end of the clavicle. Unenhanced fast-spin echo (FSE) T1-weighted imaging (T1WI) in the axial, coronal, and sagittal planes (repetition time [TR]=540 ms, echo time [TE]=11.8 ms), unenhanced FSE T2-weighted imaging in the axial plane (TR=4000 ms, TE=99 ms), contrast-enhanced (CE) T1WI in the axial and sagittal planes and fat-suppressed CE T1WI imaging in the coronal plane were performed for all patients. A contrast agent (Gd-DTPA [0.01 mmol/kg]; Magnevist, Schering) was injected intravenously. The FSE sequences using a scan plane of oblique, flip angle of 80°, number of excitations of 4, field of view of 24cm, and matrix of 24×24. The section thicknesses were 5 mm, 3 mm, and 2 mm for the axial, sagittal, and coronal planes, respectively. The section gaps were 1 mm for the axial and sagittal planes, and 0.5 mm for the coronal plane.

**Treatment**

In IMRT, 68 Gy and 60–64 Gy were delivered to the planning target volume of primary gross tumor volume and the involved lymph nodes. Clinical target volumes with high-risk, low-risk, and neck nodal regions received 60 Gy, 54 Gy, and 54 Gy, respectively. The radiation schedules were 1 fraction per day for 5 days per week. The IMRT period was 6–7 weeks. Chemotherapy regimens for CCRT were based on cisplatin, with 30–40 mg/m^2^ per week or 80–100 mg/m^2^ for 2–3 cycles in 3 weeks. IC was delivered before IMRT for 2–3 cycles within 21 days, with 80 mg/m^2^ cisplatin plus 1000 mg/m^2^ 5-fluorouracil, 75 mg/m^2^ cisplatin plus 75 mg/m^2^ docetaxel, or 60 mg/m^2^ cisplatin plus 600 mg/m^2^ 5-fluorouracil plus 60 mg/m2 docetaxel.

**Supplementary tables**

**Table A1. Incidence of skull base invasion among 1,752 nasopharyngeal carcinoma cases in two hospitals**

| Variables | Total | Hospital 1 | Hospital 2 | *p* value^*^ |
| --- | --- | --- | --- | --- |
| Pterygoid process | |  |  | 0.311 |
| None | 869(49.6%) | 664(50.3%) | 205(47.5%) |  |
| Yes | 883(50.4%) | 656(49.7%) | 227(52.5%) |  |
| Base of the sphenoid bone | |  |  | 0.001^†^ |
| None | 807(46.1%) | 577(43.7%) | 230(53.2%) |  |
| Yes | 945(53.9%) | 743(56.3%) | 202(46.8%) |  |
| Great wing of the sphenoid bone | |  |  | 0.001^†^ |
| None | 1425(81.3%) | 1033(78.3%) | 392(90.7%) |  |
| Yes | 326 (18.6%) | 287(21.7%) | 39(9.0%) |  |
| Petrous apex | |  |  | 0.689 |
| None | 1096(62.6%) | 822(62.3%) | 274(63.4%) |  |
| Yes | 656(37.4%) | 498(37.7%) | 158(36.6%) |  |
| Clivus |  |  |  | 0.864 |
| None | 1069(61.0%) | 807(61.1%) | 262(60.6%) |  |
| Yes | 683(39.0%) | 513(38.9%) | 170(39.4%) |  |
| Occipital condyle | |  |  | 0.578 |
| None | 1634(93.3%) | 1234(93.5%) | 400(92.6%) |  |
| Yes | 118(6.7%) | 86(6.5%) | 32(7.4%) |  |
| Cervical spine | |  |  | 0.345 |
| None | 1738(99.2%) | 1311(99.3%) | 427(98.8%) |  |
| Yes | 14(0.8%) | 9(0.7%) | 5(1.2%) |  |
| Paranasal sinus | |  |  | 0.003^†^ |
| None | 1310(74.8%) | 963(73%) | 347(80.3%) |  |
| Yes | 442(25.2%) | 357(27%) | 85(19.7%) |  |
| SBI |  |  |  | 0.024^†^ |
| None | 672(38.4%) | 487(36.9%) | 185(42.8%) |  |
| Yes | 1079(61.6%) | 833(63.1%) | 246(56.9%) |  |

**Abbreviation:** SBI, skull base invasion.

^*^ *p* values were calculated using Fisher’s exact test or the chi-squared test for categorical variables.

^†^*p* <0.05

**Table A2. Univariate analysis of confounding factors related to prognosis**

| Variables | OS | |  | PFS | |  | DMFS | |  | LRFS | |
| --- | --- | --- | --- | --- | --- | --- | --- | --- | --- | --- | --- |
|  | 5 years | *p* value^*^ |  | 5 years | *p* value^*^ |  | 5 years | *p* value^*^ |  | 5 years | *p* value^*^ |
| Age(years) |  | <0.001^†^ |  |  | 0.010^†^ |  |  | 0.470 |  |  | 0.660 |
| Sex |  | 0.006^†^ |  |  | 0.381 |  |  | 0.338 |  |  | 0.444 |
| Male | 83.95 |  |  | 76.16 |  |  | 85.75 |  |  | 90.29 |  |
| Female | 88.99 |  |  | 78.27 |  |  | 87.67 |  |  | 88.91 |  |
| EBV  (1×10^3^copies/ml) | | <0.001^†^ |  |  | <0.001^†^ |  |  | <0.001^†^ |  |  | 0.050 |
| <1 | 90.13 |  |  | 84.12 |  |  | 92.46 |  |  | 91.92 |  |
| <10 | 79.56 |  |  | 70.03 |  |  | 81.31 |  |  | 88.72 |  |
| ≥10 | 81.62 |  |  | 68.73 |  |  | 78.86 |  |  | 86.81 |  |
| Histologic type^※^ | | 0.269 |  |  | 0.132 |  |  | 0.496 |  |  | 0.292 |
| WHO type 1/2 | 79.4 |  |  | 68.56 |  |  | 82.71 |  |  | 85.54 |  |
| WHO type 3 | 85.49 |  |  | 77.02 |  |  | 86.4 |  |  | 90.08 |  |
| T category^‡^ | | <0.001^†^ |  |  | <0.001^†^ |  |  | <0.001^†^ |  |  | <0.001^†^ |
| T1 | 95.16 |  |  | 87.98 |  |  | 93.34 |  |  | 95.01 |  |
| T2 | 85.45 |  |  | 78.6 |  |  | 85.75 |  |  | 91.79 |  |
| T3 | 85.16 |  |  | 75.36 |  |  | 86.24 |  |  | 87.9 |  |
| T4 | 74.08 |  |  | 65.17 |  |  | 78.38 |  |  | 86.13 |  |
| N category^‡^ | | <0.001^†^ |  |  | <0.001^†^ |  |  | <0.001^†^ |  |  | <0.001^†^ |
| N0 | 93.05 |  |  | 89.77 |  |  | 95.21 |  |  | 96.08 |  |
| N1 | 87.37 |  |  | 77.5 |  |  | 87.81 |  |  | 89.57 |  |
| N2 | 75.68 |  |  | 66.69 |  |  | 77.57 |  |  | 84.5 |  |
| N3 | 65.59 |  |  | 53.83 |  |  | 65.55 |  |  | 85.76 |  |
| Stage^‡^ |  | <0.001^†^ |  |  | <0.001^†^ |  |  | <0.001^†^ |  |  | <0.001^†^ |
| I | 97.49 |  |  | 94.11 |  |  | 97.73 |  |  | 97.17 |  |
| II | 94.24 |  |  | 87.01 |  |  | 93.25 |  |  | 94.03 |  |
| III | 85.83 |  |  | 76.24 |  |  | 86.43 |  |  | 88.36 |  |
| IV | 73.33 |  |  | 63.78 |  |  | 76.58 |  |  | 86.21 |  |
| Chemotherapy | | 0.571 |  |  | 0.167 |  |  | 0.026^†^ |  |  | 0.068 |
| No | 86.22 |  |  | 79.93 |  |  | 90.56 |  |  | 93.48 |  |
| Yes | 85.1 |  |  | 76.06 |  |  | 85.4 |  |  | 89.23 |  |
| IC |  | 0.006^†^ |  |  | <0.001^†^ |  |  | <0.001^†^ |  |  | 0.017^†^ |
| No | 87.83 |  |  | 80.59 |  |  | 89.89 |  |  | 91.88 |  |
| Yes | 82.72 |  |  | 72.92 |  |  | 82.71 |  |  | 87.99 |  |

**Abbreviations:** AJCC, American Joint Committee on Cancer; EBV, Epstein-Barr virus; IC, induction chemotherapy; OS, overall survival; PFS, progression-free survival; DMFS, distant metastasis-free survival; LRFS, locoregional recurrence-free survival; WHO, World Health Organization.

^*^ *p* values were calculated using the log-rank test.

^†^*p* <0.05

※According to the 2005 World Health Organization classification of tumors.

‡According to the 8th edition of the AJCC staging system.

**Table A3.** **Numbers of acute adverse events during treatment for patients with nasopharyngeal carcinoma at Hospital 1**

| Variables | Hospital 1, N=1320 | | χ2 | slight SBI, N=174 | | χ2 | severe SBI, N=358 | | χ2 |
| --- | --- | --- | --- | --- | --- | --- | --- | --- | --- |
|  | IC | non_IC | *p* value^*a^ | IC | non_IC | *p* value^*b^ | IC | non_IC | *p* value^*c^ |
| Hematological | |  |  |  |  |  |  |  |  |
| Neutropenia |  |  | <0.001^†^ |  |  | <0.001^†^ |  |  | <0.001^†^ |
| None | 116(17.6%) | 364(55.2%) | | 11(16.2%) | 67(63.2%) |  | 30(14.9%) | 77(49%) |  |
| Grade 1/2 | 273(41.4%) | 254(38.5%) | | 32(47.1%) | 33(31.1%) |  | 91(45.3%) | 65(41.4%) |  |
| Grade 3/4 | 271(41.1%) | 42(6.4%) |  | 25(36.8%) | 6(5.7%) |  | 80(39.8%) | 15(9.6%) |  |
| Febrile neutropenia |  |  | 1 | 0 | 0 | - | 0 | 0 | - |
| None | 659(99.8%) | 660(100%) | |  |  |  |  |  |  |
| Grade 1/2 | 1(0.2%) | 0 |  |  |  |  |  |  |  |
| Grade 3/4 | 0 | 0 |  |  |  |  |  |  |  |
| Neutropenic infection | 0 | 0 | - | 0 | 0 | - | 0 | 0 | - |
| Leucopenia |  |  | <0.001^†^ |  |  | 0.017^†^ |  |  | <0.001^†^ |
| None | 45(6.8%) | 167(25.3%) | | 6(8.8%) | 28(26.4%) |  | 11(5.5%) | 35(22.3%) |  |
| Grade 1/2 | 318(48.2%) | 360(54.5%) | | 38(55.9%) | 50(47.2%) |  | 101(50.2%) | 88(56.1%) |  |
| Grade 3/4 | 297(45%) | 133(20.2%) | | 24(35.3%) | 28(26.4%) |  | 89(44.3%) | 34(21.7%) |  |
| Anemia |  |  | <0.001^†^ |  |  | 0.007^†^ |  |  | <0.001^†^ |
| None | 39(5.9%) | 174(26.4%) | | 7(10.3%) | 32(30.2%) |  | 9(4.5%) | 33(21%) |  |
| Grade 1/2 | 575(87.1%) | 471(71.4%) | | 58(85.3%) | 69(65.1%) |  | 181(90%) | 120(76.4%) | |
| Grade 3/4 | 46(7%) | 15(2.3%) |  | 3(4.4%) | 5(4.7%) |  | 11(5.5%) | 4(2.5%) |  |
| Thrombocytopenia |  |  | <0.001^†^ |  |  | 0.534 |  |  | 0.467 |
| None | 417(63.2%) | 531(80.5%) | | 48(70.6%) | 82(77.4%) |  | 134(66.7%) | 114(72.6%) | |
| Grade 1/2 | 193(29.2%) | 105(15.9%) | | 17(25%) | 19(17.9%) |  | 54(26.9%) | 34(21.7%) |  |
| Grade 3/4 | 50(7.6%) | 24(3.6%) |  | 3(4.4%) | 5(4.7%) |  | 13(6.5%) | 9(5.7%) |  |
| Lymphopenia |  |  | 0.287^†^ |  |  | 0.714 |  |  | 0.401 |
| None | 17(2.6%) | 42(6.4%) |  | 6(8.8%) | 6(5.7%) |  | 2(1%) | 3(1.9%) |  |
| Grade 1/2 | 119(18%) | 119(18%) |  | 14(20.6%) | 21(19.8%) |  | 32(15.9%) | 18(11.5%) |  |
| Grade 3/4 | 524(79.4%) | 499(75.6%) | | 48(70.6%) | 79(74.5%) |  | 167(83.1%) | 136(86.6%) | |
| Non-hematological | |  |  |  |  |  |  |  |  |
| Stomatitis (mucositis) |  |  | 0.399 |  |  | 0.146 |  |  | 0.107 |
| None | 120(18.2%) | 133(20.2%) | | 12(17.6%) | 24(22.6%) |  | 29(14.4%) | 31(19.7%) |  |
| Grade 1/2 | 411(62.3%) | 387(58.6%) | | 47(69.1%) | 58(54.7%) |  | 132(65.7%) | 86(54.8%) |  |
| Grade 3/4 | 129(19.5%) | 140(21.2%) | | 9(13.2%) | 24(22.6%) |  | 40(19.9%) | 40(25.5%) |  |
| Vomiting or nausea |  |  | <0.001^†^ |  |  | 0.002^†^ |  |  | <0.001^†^ |
| None | 133(20.2%) | 314(47.6%) | | 14(20.6%) | 49(46.2%) |  | 36(17.9%) | 64(40.8%) |  |
| Grade 1/2 | 508(77%) | 329(49.8%) | | 53(77.9%) | 54(50.9%) |  | 161(80.1%) | 89(56.7%) |  |
| Grade 3/4 | 19(2.9%) | 17(2.6%) |  | 1(1.5%) | 3(2.8%) |  | 4(2%) | 4(2.5%) |  |
| Dry mouth |  |  | 0.09 |  |  | 0.269 |  |  | 1 |
| None | 386(58.5%) | 355(53.8%) | | 43(63.2%) | 58(54.7%) |  | 105(52.2%) | 82(52.2%) |  |
| Grade 1/2 | 274(41.5%) | 305(46.2%) | | 25(36.8%) | 48(45.3%) |  | 96(47.8%) | 75(47.8%) |  |
| Grade 3/4 | 0 | 0 |  | 0 | 0 |  | 0 | 0 |  |
| Diarrhea |  |  | <0.001^†^ |  |  | 0.031^†^ |  |  | <0.001^†^ |
| None | 582(88.2%) | 644(97.6%) | | 60(88.2%) | 103(97.2%) | | 176(87.6%) | 155(98.7%) | |
| Grade 1/2 | 71(10.8%) | 16(2.4%) |  | 7(10.3%) | 3(2.8%) |  | 24(11.9%) | 2(1.3%) |  |
| Grade 3/4 | 7(1.1%) | 0 |  | 1(1.5%) | 0 |  | 1(0.5%) | 0 |  |
| Skin |  |  | 0.317 |  |  | 1 |  |  | 0.08 |
| None | 189(28.6%) | 166(25.2%) | | 19(27.9%) | 30(28.3%) |  | 62(30.8%) | 32(20.4%) |  |
| Grade 1/2 | 461(69.8%) | 481(72.9%) | | 48(70.6%) | 75(70.8%) |  | 135(67.2%) | 121(77.1%) | |
| Grade 3/4 | 10(1.5%) | 13(2%) |  | 1(1.5%) | 1(0.9%) |  | 4(2%) | 4(2.5%) |  |
| Hair loss |  |  | <0.001^†^ |  |  | 0.038^†^ |  |  | 0.037^†^ |
| None | 535(81.1%) | 625(94.7%) | | 58(85.3%) | 100(94.3%) | | 165(82.1%) | 142(90.4%) | |
| Grade 1/2 | 117(17.7%) | 33(5%) |  | 10(14.7%) | 5(4.7%) |  | 33(16.4%) | 15(9.6%) |  |
| Grade 3/4 | 8(1.2%) | 2(0.3%) |  | 0(0%) | 1(0.9%) |  | 3(1.5%) | 0 |  |
| Fatigue |  |  | 0.106 |  |  | 0.786 |  |  | 0.5 |
| None | 579(87.7%) | 598(90.6%) | | 63(92.6%) | 96(90.6%) |  | 175(87.1%) | 141(89.8%) | |
| Grade 1/2 | 81(12.3%) | 62(9.4%) |  | 5(7.4%) | 10(9.4%) |  | 26(12.9%) | 16(10.2%) |  |
| Grade 3/4 | 0 | 0 |  | 0 | 0 |  | 0 | 0 |  |
| Infection or fever |  |  | <0.001^†^ |  |  | 0.294 |  |  | 0.007^†^ |
| None | 621(94.1%) | 652(98.8%) | | 65(95.6%) | 105(99.1%) | | 189(94%) | 156(99.4%) | |
| Grade 1/2 | 37(5.6%) | 8(1.2%) |  | 3(4.4%) | 1(0.9%) |  | 12(6%) | 1(0.6%) |  |
| Grade 3/4 | 2(0.3%) | 0 |  | 0 | 0 |  | 0 | 0 |  |
| Allergic reaction |  |  | 0.413 |  |  | 0.426 |  |  | 1 |
| None | 631(95.6%) | 641(97.1%) | | 64(94.1%) | 103(97.2%) | | 194(96.5%) | 152(96.8%) | |
| Grade 1/2 | 28(4.2%) | 18(2.7%) |  | 4(5.9%) | 3(2.8%) |  | 6(3%) | 5(3.2%) |  |
| Grade 3/4 | 1(0.2%) | 1(0.2%) |  | 0 | 0 |  | 1(0.5%) | 0 |  |
| Deafness or otitis |  |  | 0.517 |  |  | 0.469 |  |  | 0.032^†^ |
| None | 570(86.4%) | 574(87%) |  | 58(85.3%) | 95(89.6%) |  | 180(89.6%) | 128(81.5%) | |
| Grade 1/2 | 88(13.3%) | 86(13%) |  | 10(14.7%) | 11(10.4%) |  | 20(10%) | 29(18.5%) |  |
| Grade 3/4 | 2(0.3%) | 0 |  | 0 | 0 |  | 1(0.5%) | 0 |  |
| Esophagus discomfort |  |  | 0.14 |  |  | 0.515 |  |  | 0.091 |
| None | 288(43.6%) | 253(38.3%) | | 29(42.6%) | 46(43.4%) |  | 91(45.3%) | 55(35%) |  |
| Grade 1/2 | 351(53.2%) | 382(57.9%) | | 38(55.9%) | 55(51.9%) |  | 105(52.2%) | 94(59.9%) |  |
| Grade 3/4 | 21(3.2%) | 25(3.8%) |  | 1(1.5%) | 5(4.7%) |  | 5(2.5%) | 8(5.1%) |  |
| Throat discomfort |  |  | 0.25 |  |  | 1 |  |  | 0.495 |
| None | 538(81.5%) | 530(80.3%) | | 57(83.8%) | 89(84%) |  | 163(81.1%) | 123(78.3%) | |
| Grade 1/2 | 122(18.5%) | 127(19.2%) | | 11(16.2%) | 17(16%) |  | 38(18.9%) | 33(21%) |  |
| Grade 3/4 | 0 | 3(0.5%) |  | 0 | 0 |  | 0 | 1(0.6%) |  |
| Nephrotoxic event |  |  | 0.644 |  |  | 0.104 |  |  | 0.513 |
| None | 638(96.7%) | 645(97.7%) | | 63(92.6%) | 104(98.1%) | | 197(98%) | 152(96.8%) | |
| Grade 1/2 | 21(3.2%) | 14(2.1%) |  | 5(7.4%) | 2(1.9%) |  | 4(2%) | 5(3.2%) |  |
| Grade 3/4 | 1(0.2%) | 1(0.2%) |  | 0 | 0 |  | 0 | 0 |  |
| Hepatotoxic event |  |  | 0.001^†^ |  |  | 0.248 |  |  | 0.138 |
| None | 551(83.5%) | 599(90.8%) | | 55(80.9%) | 95(89.6%) |  | 164(81.6%) | 140(89.2%) | |
| Grade 1/2 | 84(12.7%) | 47(7.1%) |  | 11(16.2%) | 8(7.5%) |  | 27(13.4%) | 13(8.3%) |  |
| Grade 3/4 | 25(3.8%) | 14(2.1%) |  | 2(2.9%) | 3(2.8%) |  | 10(5%) | 4(2.5%) |  |
| Digestive discomfort |  |  | <0.001^†^ |  |  | <0.001^†^ |  |  | 0.001^†^ |
| None | 82(12.4%) | 209(31.7%) | | 6(8.8%) | 37(34.9%) |  | 21(10.4%) | 38(24.2%) |  |
| Grade 1/2 | 530(80.3%) | 429(65%) |  | 60(88.2%) | 64(60.4%) |  | 165(82.1%) | 114(72.6%) | |
| Grade 3/4 | 48(7.3%) | 22(3.3%) |  | 2(2.9%) | 5(4.7%) |  | 15(7.5%) | 5(3.2%) |  |
| Cardiac discomfort |  |  | 0.055 |  |  | 0.558 |  |  | 0.513 |
| None | 640(97%) | 651(98.6%) | | 66(97.1%) | 105(99.1%) | | 197(98%) | 152(96.8%) | |
| Grade 1/2 | 20(3%) | 9(1.4%) |  | 2(2.9%) | 1(0.9%) |  | 4(2%) | 5(3.2%) |  |
| Grade 3/4 | 0 | 0 |  | 0 | 0 |  | 0 | 0 |  |

**Abbreviations:** IC, induction chemotherapy; slight SBI, T3 patients with pterygoid process and/or base of the sphenoid bone invasion only; severe SBI, T3 patients with other SBIs.

^*^ *p* values were calculated for acute adverse events distribution between patients with nasopharyngeal carcinoma treated with and without induction chemotherapy in hospital 1, ^*a^ total patient in hospital 1, ^*b^ slight SBI group in hospital, ^*c^ severe SBI group in hospital 1, using Fisher’s exact test or the chi-squared test for categorical variables.

†*p* <0.05

**Note1.** the acute adverse events were graded according to the Common Terminology Criteria for Adverse Events (CTCAE) Version 4.0.**Table A4. Prognosis in the 8^th^ edition AJCC T staging system and proposed T category.**

| Variables | number |  |  | OS | |  | PFS | | | |
| --- | --- | --- | --- | --- | --- | --- | --- | --- | --- | --- |
|  |  | surv. | *p* value^*^ | HR (95% CI)^†^ | *p* value^*,‡^ | | surv. | *p* value^*^ | HR (95% CI)^†^ | *p* value^*,‡^ |
| **New T** |  |  |  |  |  |  |  |  |  |  |
| T1 | 451 | 95.20% |  | reference |  |  | 88.00% |  | reference |  |
| newT2 | 437 | 88.90% | <0.001^§^ | 2.28 (1.37–3.79) | 0.00^§^ |  | 80.90% | 0.01^§^ | 1.6 (1.13–2.26) | 0.01^§^ |
| newT3 | 454 | 81.50% | 0.00^§^ | 3.91 (2.43–6.28) | 0.00^§^ |  | 71.50% | 0.00^§^ | 2.53 (1.84–3.49) | 0.00^§^ |
| T4 | 410 | 74.10% | 0.01^§^ | 5.99 (3.78–9.51) | 0.00^§^ |  | 65.20% | 0.04^§^ | 3.27 (2.38–4.48) | 0.00^§^ |
| **AJCC 8th** |  |  |  |  |  |  |  |  |  |  |
| T1 | 451 | 95.20% |  | reference |  |  | 88.00% |  | reference |  |
| T2 | 213 | 85.50% | <0.001^§^ | 2.89 (1.65–5.05) | 0.00^§^ |  | 78.60% | 0.00^§^ | 1.78 (1.19–2.66) | 0.00^§^ |
| T3 | 678 | 85.20% | 0.70 | 3.14 (1.97–5.00) | 0.00^§^ |  | 75.40% | 0.28 | 2.14 (1.57–2.92) | 0.00^§^ |
| T4 | 410 | 74.10% | <0.001^§^ | 5.99 (3.78–9.51) | 0.00^§^ |  | 65.20% | <0.001^§^ | 3.27 (2.38–4.48) | 0.00^§^ |
| (continued) |  |  |  |  |  |  |  |  |  |  |
| Variables | number | DMFS | | | |  | LRFS | | | |
|  |  | surv. | *p* value^*^ | HR (95% CI) ^†^ | *p* value^*,‡^ | | surv. | *p* value^*^ | HR (95% CI) ^†^ | *p* value^*,‡^ |
| **New T** |  |  |  |  |  |  |  |  |  |  |
| T1 | 451 | 93.30% |  | reference |  |  | 95.00% |  | reference |  |
| newT2 | 437 | 87.80% | 0.01^§^ | 1.81 (1.15–2.85) | 0.01^§^ |  | 91.40% | 0.07 | 1.64 (0.97–2.77) | 0.07 |
| newT3 | 454 | 84.40% | 0.15 | 2.38 (1.54–3.67) | 0.00^§^ |  | 86.30% | 0.04^§^ | 2.58 (1.58–4.20) | 0.00^§^ |
| T4 | 410 | 78.40% | 0.02^§^ | 3.48 (2.29–5.29) | 0.00^§^ |  | 86.10% | 0.89 | 2.65 (1.61–4.36) | 0.00^§^ |
| **AJCC 8^th^** |  |  |  |  |  |  |  |  |  |  |
| T1 | 451 | 93.30% |  | reference |  |  | 95.00% |  | reference |  |
| T2 | 213 | 85.80% | 0.00^§^ | 2.16 (1.3–3.60) | 0.00^§^ |  | 91.80% | 0.20 | 1.53 (0.81–2.89) | 0.19 |
| T3 | 678 | 86.20% | 0.84 | 2.07 (1.36–3.14) | 0.00^§^ |  | 87.90% | 0.14 | 2.29 (1.43–3.66) | 0.00^§^ |
| T4 | 410 | 78.40% | <0.001^§^ | 3.48 (2.29–5.28) | 0.00^§^ |  | 85.10% | 0.42 | 2.65 (1.61–4.36) | 0.00^§^ |

**Abbreviation**s: AJCC, American Joint Committee on Cancer; CI, confidence interval; OS, overall survival; PFS, progression-free survival; DMFS, distant metastasis-free survival; LRFS, locoregional recurrence-free survival; HR, hazard ratio; surv., survival rate.

^*^ *p* values were calculated using the log rank test and were compared between the previous T category and the next T category, such as T1 vs. newT2, newT2 vs. newT3, newT3 vs. T4, etc.

^†,‡^ hazard ratio (HR), and *p* values were calculated using multivariate Cox regression analysis.

^§^*p* <0.05

**Note1.** Survival curves are shown in **Fig. 4** and **Fig. A4.**

**Table A5. C-index of the proposed T staging system and 8^th^ edition AJCC staging system in patients with NPC**

|  | C-index (95% CI) | OS | |  | | PFS | | |  | DMFS | |  | LRFS | |
| --- | --- | --- | --- | --- | --- | --- | --- | --- | --- | --- | --- | --- | --- | --- |
|  |  | train | test | |  | | train | test |  | train | test |  | train | test |
| T | Proposed T | 0.672  (0.635–0.709) | 0.661  (0.604–0.719) | |  | | 0.617  (0.585–0.648) | 0.637  (0.586–0.687) |  | 0.615  (0.576–0.654) | 0.640  (0.572–0.707) |  | 0.598  (0.549–0.647) | 0.613  (0.544–0.682) |
|  | 8^th^ edition T | 0.658  (0.620–0.695) | 0.642  (0.584–0.701) | |  | | 0.607  (0.576–0.638) | 0.624  (0.572–0.675) |  | 0.607  (0.568–0.646) | 0.638  (0.571–0.705) |  | 0.597  (0.549–0.644) | 0.538  (0.445–0.630) |
|  | *p* value^†^ | 0.000^*^ | 0.000^*^ | |  | | 0.000^*^ | 0.001^*^ |  | 0.009^*^ | 0.101 |  | 0.470 | 0.000^*^ |
| T  + cf | Proposed T | 0.751  (0.716–0.786) | 0.725  (0.672–0.779) | |  | | 0.679  (0.648–0.710) | 0.688  (0.638–0.737) |  | 0.712  (0.675–0.748) | 0.689  (0.627–0.751) |  | 0.637  (0.586–0.687) | 0.672  (0.608–0.735) |
|  | 8^th^ edition T | 0.747  (0.711–0.782) | 0.713  (0.658–0.768) | |  | | 0.677  (0.646–0.708) | 0.680  (0.629–0.731) |  | 0.713  (0.676–0.749) | 0.685  (0.622–0.747) |  | 0.645  (0.596–0.694) | 0.627  (0.552–0.703) |
|  | *p* value^†^ | 0.122 | 0.046 ^*^ | |  | | 0.889 | 0.429 |  | 0.445 | 0.887 |  | 0.346 | 0.086 |

**Abbreviations**: AJCC, American Joint Committee on Cancer; C-index, Harrel concordance index; cf, confounding factors; CI, confidence interval; NPC, nasopharyngeal carcinoma; OS, overall survival; PFS, progression-free survival; DMFS, distant metastasis-free survival; LRFS, locoregional recurrence-free survival; train, hospital 1; test, hospital 2.

^*^ *p* <0.05

^†^ *p* values of C-index were calculated using log rank test.

**Supplementary Figures**


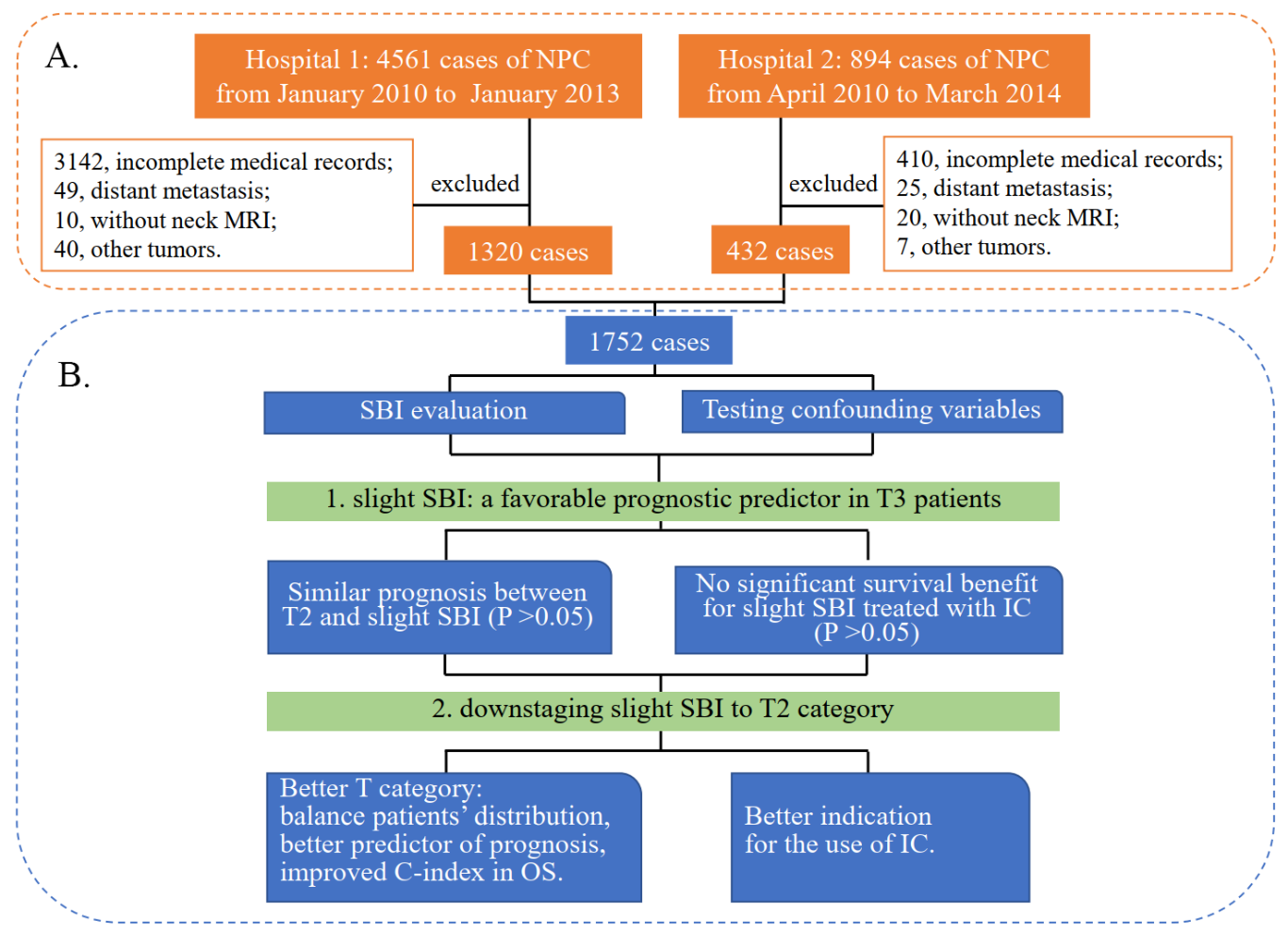


**Fig. A1. Flowchart of the study.** Patients recruitment (A) and statistical analysis (B). First, we confirmed the feasibility of downstaging NPC patients with slight SBI to the T2 category by comparing the prognosis between SBI subclassification, slight SBI and T2 patients, and patients treated with or without IC. Second, we explored the strengths of the proposed T category by investigating its prognostic value and the implications of IC administration.

**Abbreviations:** C-index, Harrel concordance index; IC, induction chemotherapy; NPC, nasopharyngeal carcinoma; OS, overall survival; SBI, skull base invasion.


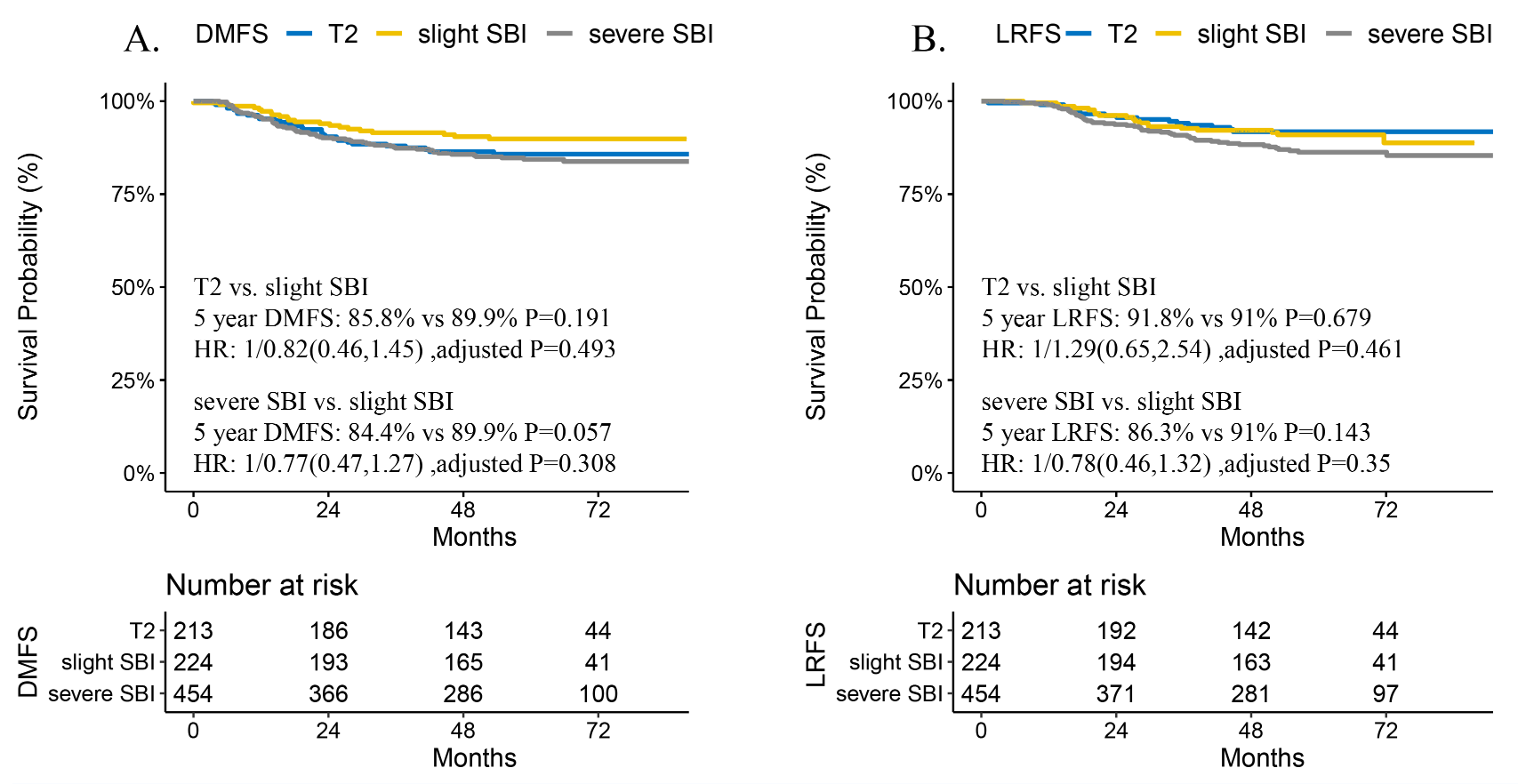


**Fig. A2. Prognosis for DMFS and LRFS in slight SBI patients compared with T2 and severe SBI patients.** DMFS (A) and LRFS (B) were slightly higher in the slight SBI group than those in the severe SBI group but failed to achieve statistical significance; no statistical differences were observed for DMFS and LRFS between the slight SBI and T2 groups.

**Abbreviations**: DMFS, distant metastasis-free survival; HR, hazard ratio; LRFS, locoregional recurrence-free survival; SBI, skull base invasion.

**Note 1.** Detailed results are presented in **Table 2**.


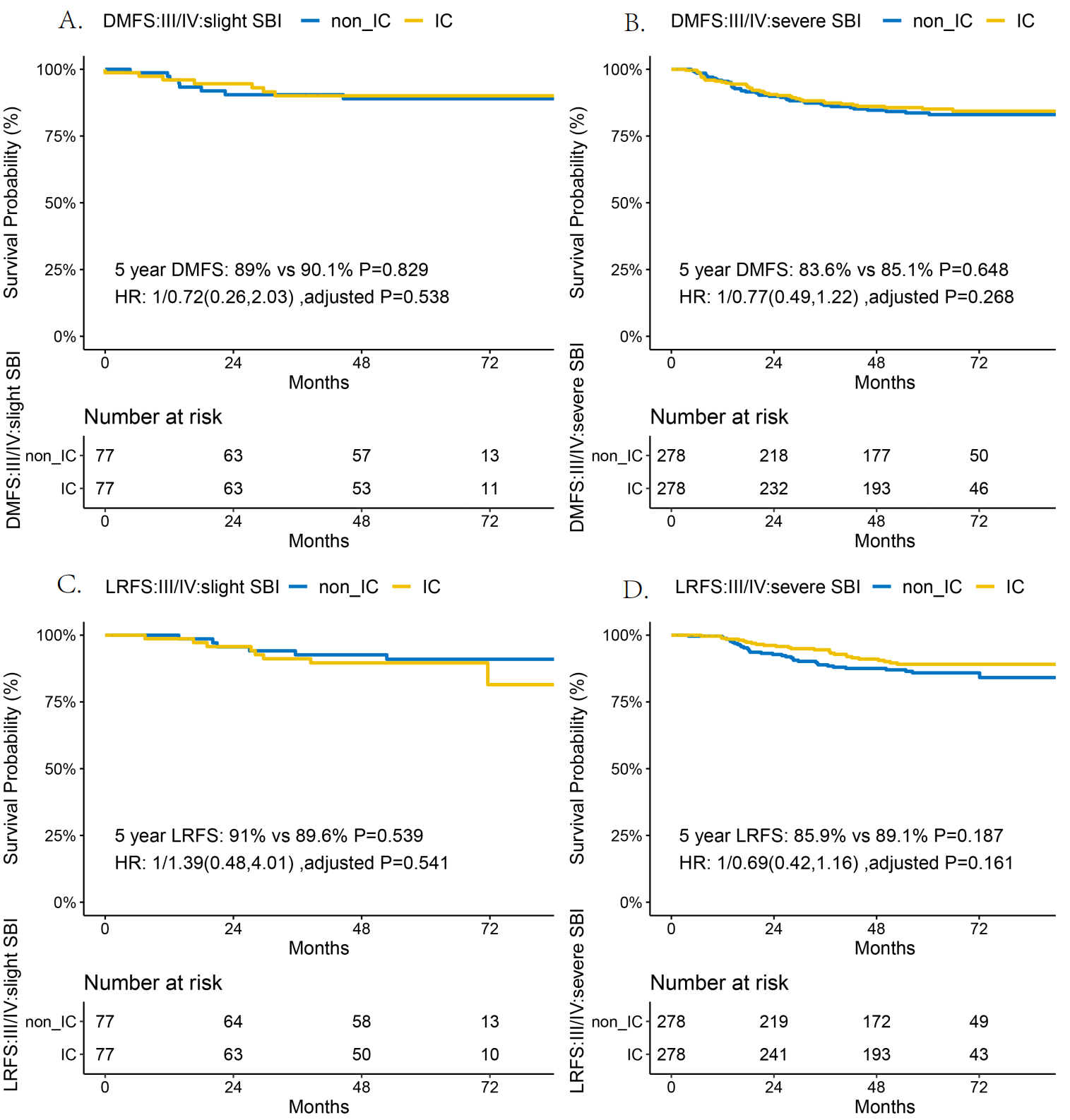


**Fig. A3. Survival outcomes for DMFS and LRFS among SBI subclassification treated with or without IC.** Among patients with stage III/IV NPC, the 5-year DMFS (A, B) and LRFS (C, D) almost overlapped for different SBI subclassifications treated with or without additional IC.

**Abbreviations:** DMFS, distant metastasis-free survival; HR, hazard ratio; IC, induction chemotherapy; LRFS, locoregional recurrence-free survival; SBI, skull base invasion.


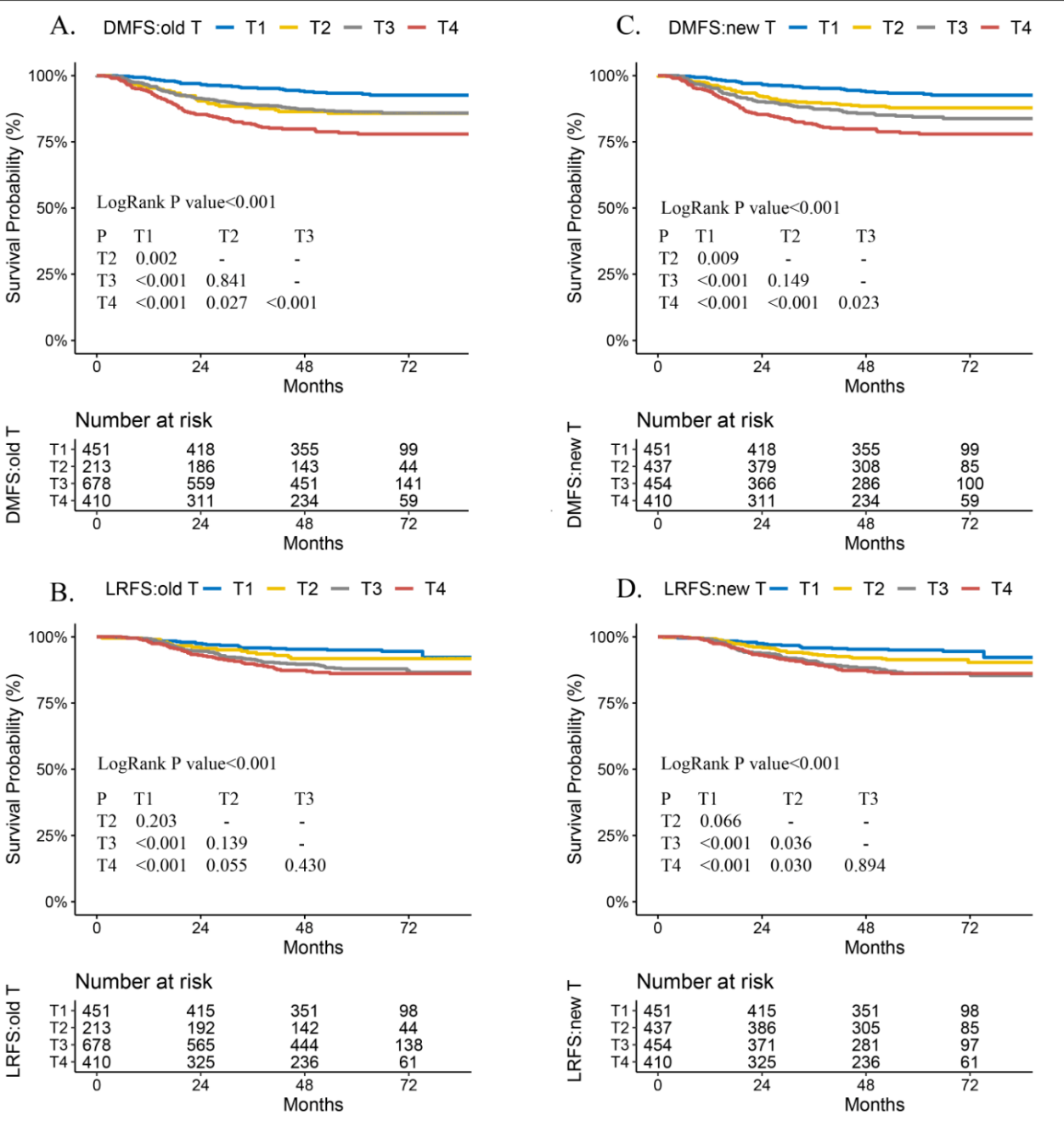


**Fig.A4** **Prognosis for DMFS and LRFS in the 8^th^ edition AJCC T staging system and proposed T category.** In the 8^th^ edition AJCC T staging system, the DMFS (A) and LRFS (B) for T2 and T3 categories almost overlapped; After downstaging slight SBI group from T3 to T2 category, significant separated prognosis was observed in the LRFS of proposed T category (D).

**Abbreviations:** AJCC, American Joint Committee on Cancer; HR, hazard ratio; IC, induction chemotherapy; DMFS, distant metastasis-free survival; LRFS, locoregional recurrence-free survival; SBI, skull base invasion.


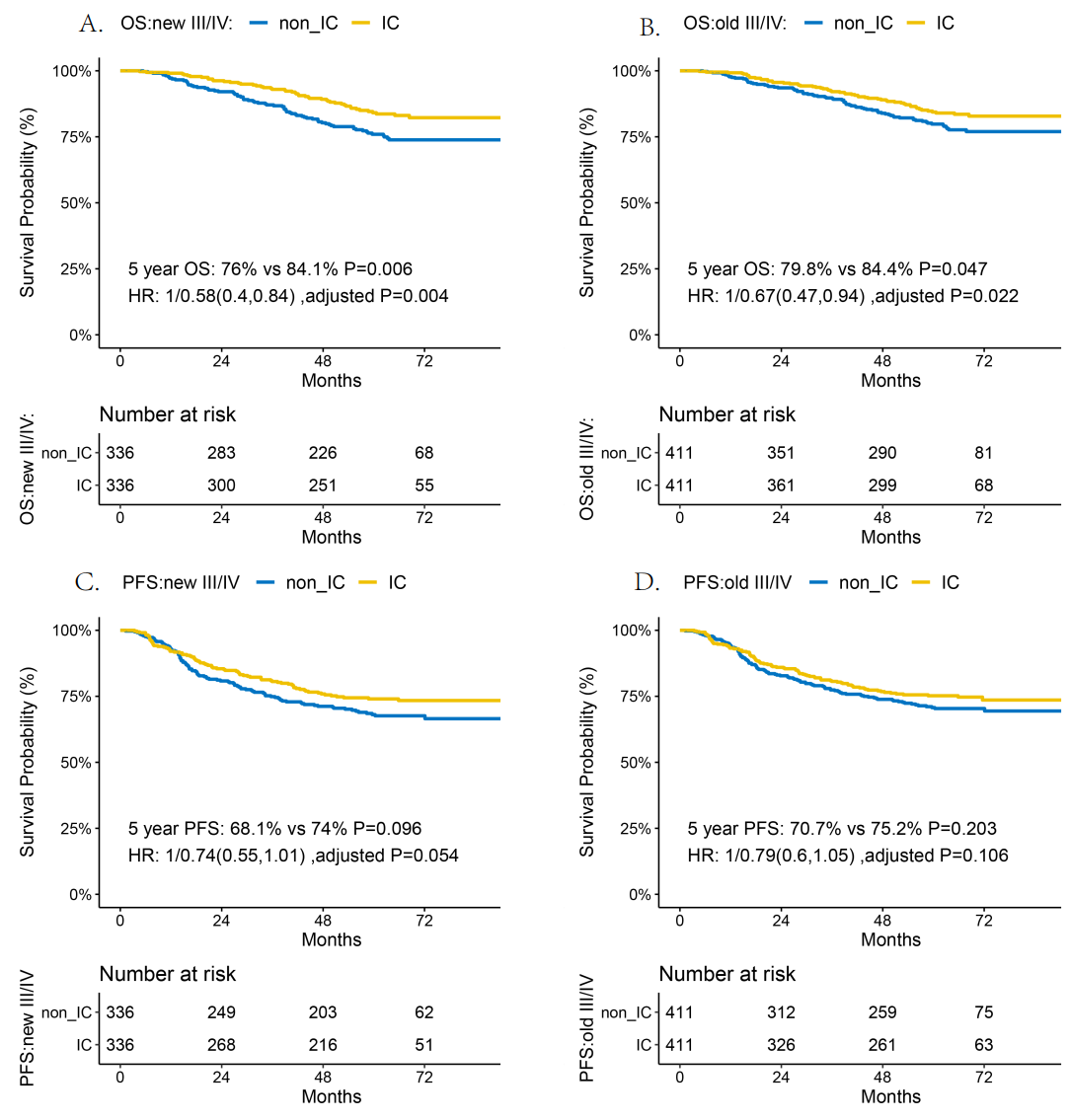


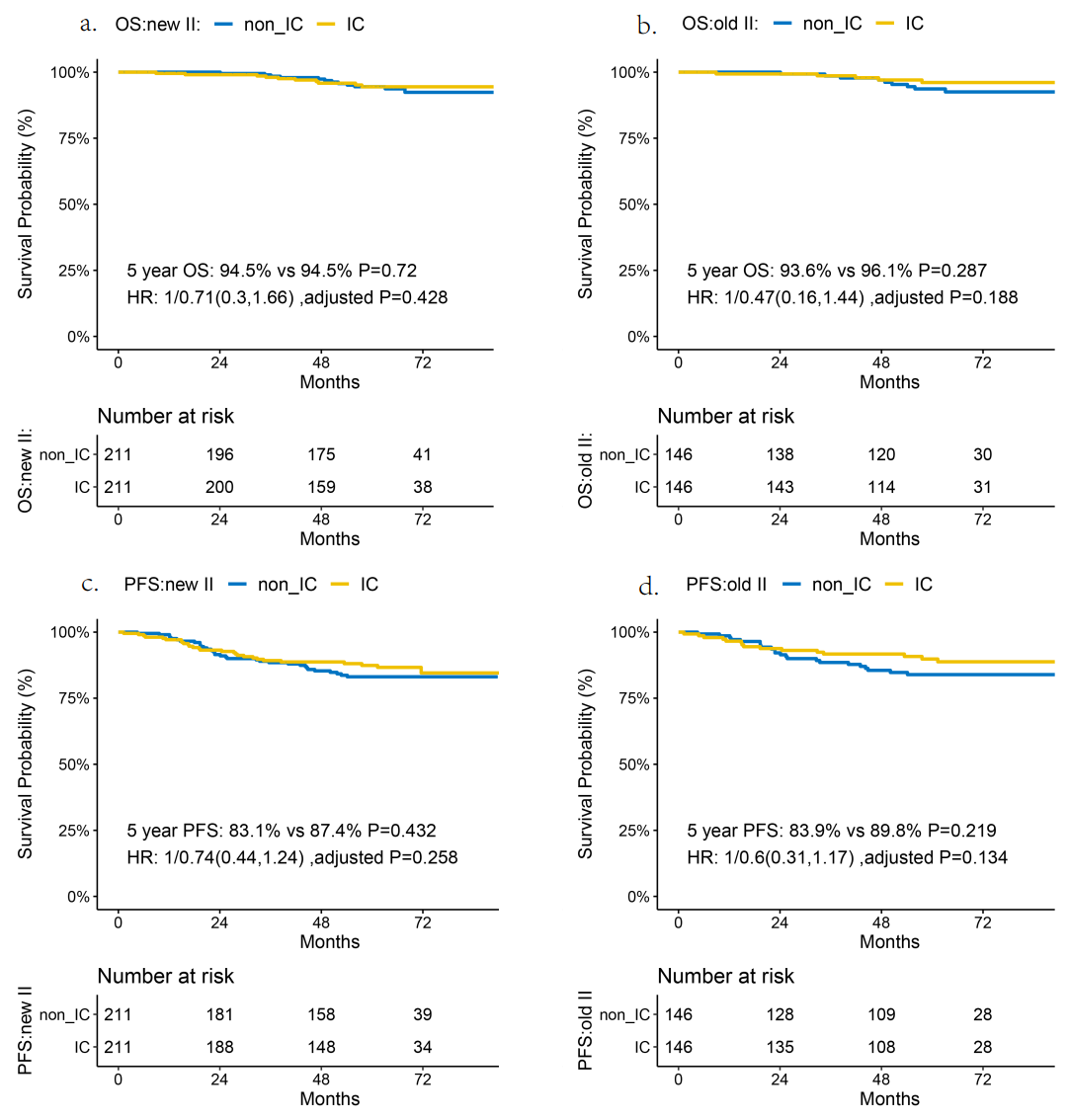


**Fig. A5. The implication of the proposed T category in the use of IC.** New tumor stage was obtained according to the proposed T category. OS in patients treated with IC improved significantly, which is more obvious in the new Ⅲ/Ⅳ stage (A) than in the conventional Ⅲ/Ⅳ stage (B). Improved PFS in patients treated with IC was obvious in the new Ⅲ/Ⅳ stage (C) and was close to statistical significance, while it was no significant differences in conventional Ⅲ/Ⅳ stage (D). For NPC patients in Ⅱ stage, patients gained no survival benefit from the use of IC in both the new and conventional 8^th^ tumor stage (a-d).

**Abbreviations:** HR, hazard ratio; IC, induction chemotherapy; NPC, nasopharyngeal carcinoma; OS, overall survival; PFS, progression-free survival.
